# Magnetic field based investigation of Brugada syndrome

**DOI:** 10.1007/s00249-026-01826-7

**Published:** 2026-03-04

**Authors:** Martina Nicoletti, Anna Crispino, Alessandro Loppini, Alessio Gizzi, Letizia Chiodo, Christian Cherubini, Simonetta Filippi

**Affiliations:** 1 Department of Engineering, Università Campus Bio-Medico di Roma, Via Alvaro del Portillo 21, Rome, 00128 Italy; 2 Center for Life Nano & Neuro Science, Istituto Italiano di Tecnologia, Viale Regina Elena, 291, Rome, 00161 Italy; 3 Department of Medicine and Surgery, Università Campus Bio-Medico di Roma, Via Alvaro del Portillo 21, Rome, 00128 Italy; 4 Department of Science and Bio-Technology, Università Campus Bio-Medico di Roma, Via Alvaro del Portillo 21, Rome, 00128 Italy; 5 Istituto Nazionale di Ottica, Consiglio Nazionale delle Ricerche, Largo Enrico Fermi, Florence, 50125 Italy; 6 ICRANet - International Center for Relativistic Astrophysics Network, Piazza della Repubblica, 10, Pescara, 65122 Italy

**Keywords:** Cardiac alternans, Magnetic-field, Brugada, Magnetocardiogram

## Abstract

Brugada syndrome (BrS) is an inherited cardiac disorder associated with and increased risk of sudden cardiac death. The syndrome is associated with a complex current distribution and repolarization dispersion. The traditional methods for the diagnosis rely on standard electrocardiographic (ECG) assessment of the cardiac dynamics. Recent advancements in magnetocardiography suggest that the magnetic-field based investigation of cardiac electrophysiology could offer novel perspectives for the investigation of current abnormalities in BrS patients which go beyond the conventional ECG capabilities to capture intricate current distributions. In this work, we propose a framework for the magnetic analysis of action potential disturbances in Brugada syndrome. We exploit a 1D reduced-order cable geometry for analyzing the relation between the pathological electrical activity in saddleback and coved type BrS and the corresponding magnetic field. Also, we compute transmural pseudo-magnetocardiograms and demonstrate their capabilities of detecting both types of BrS. Overall, our result highlight the potential of magnetic-field oriented investigation of cardiac disturbances related to BrS.

## Introduction

Cardiac arrhythmias are among the major causes of sudden cardiac death (SCD), significantly impacting global mortality rates (Di Cesare et al. 2024; Zheng et al. 2001; Kim et al. 2011). Arrhythmias have a broad spectrum of causes, including lifestyle, comorbidities and genetic factors (Keating and Sanguinetti 2001; Shantsila et al. 2024), as in the case of Long-QT syndrome, Catecholaminergic Polymorphic Ventricular Tachycardia (CPVT), and Brugada syndrome (BrS) (Keating and Sanguinetti 2001; Antzelevitch et al. 2005). BrS is a genetic disorder associated to an increased risk of ventricular fibrillation and SCD (Antzelevitch et al. 2005; Mizusawa and Wilde 2012; Sieira et al. 2016), characterized by ST-segment elevation in the right precordial and by T-wave inversion in electrocardiograms (ECGs) (Antzelevitch et al. 2005; Mizusawa and Wilde 2012; Sieira et al. 2016; Cutler et al. 2024). Until today, BrS has been linked to mutations of the *SCN5A* gene encoding for the $\alpha -$subunit of the cardiac sodium channel Na_v_1.5, but also to dysfunctions in the outward rectifier, delayed rectifier, and ATP-sensitive potassium channels (Cutler et al. 2024; Sieira et al. 2016; Tse et al. 2016). Besides the genetic origin, the pathophysiology of the syndrome has been tentatively explained hypothesizing two main mechanisms: depolarization and repolarization disorders (Cutler et al. 2024; Sieira et al. 2016; Tse et al. 2016). In the first scenario, the reduced sodium currents give rise to a slower upstroke and to a reduction of the conduction velocity leading to arrhythmogenesis (Tse et al. 2016). Instead, the repolarization hypothesis explains the syndrome based on alteration in the transient outward rectifier current, causing an heterogeneous reduction of the action potential (AP) dome (Mizusawa and Wilde 2012; Cutler et al. 2024; Tse et al. 2016). Beyond traditional electrical measurement techniques, the analysis of cardiac magnetic fields has emerged as a complementary diagnostic approach for identifying subtle electrophysiological abnormalities (Roth et al. 1988; Roth 2024, 2023; Plonsey 1982; Holzer et al. 2004; Lachlan et al. 2024; Brisinda et al. 2023). However, a major challenge in magnetocardiographic studies is the extremely low amplitude of cardiac magnetic fields, which typically fall within the pT-nT range, thereby requiring highly sensitive detection methodologies (Baudenbacher et al. 2002; Nakayama and Uchiyama 2015; Webb et al. 2021). Recent advancements in quantum magnetometry, particularly the development of Superconducting Quantum Interference Devices (SQUIDs) and Nitrogen-Vacancy (NV) centers in diamonds, have substantially enhanced the resolution of biomagnetic measurements. These innovations facilitate the detection of weak magnetic signals at the cellular scale, offering new possibilities for high-precision electrophysiological assessments the molecular and biophysical mechanisms underlying arrhythmias in BrS and other channelopathies (Barry et al. 2016, 2020; Arai et al. 2022; Fenici et al. 2025; Yu et al. 2024). Moreover, prior studies have shown how the analysis of the magnetic activity of tissues and organs can highlight electrically silent but magnetically active components, which are not detectable with classical investigation tools based on the measurement of the electrical activity such as the electrocardiogram (EGC) (Roth et al. 1988; Irimia et al. 2009; Wikswo and Barach 1982; Roth and Wikswo 1986; Fischer et al. 1999; Holzer et al. 2004; McBride et al. 2010).

Mathematical modeling has been proved a powerful instrument in elucidating the complex dynamics underlying cardiac arrhythmias, offering insights into their initiation and evolution (Fenton and Karma 1998; Gizzi et al. 2017; Filippi et al. 2014). In this work we perform an investigation of BrS in 1D homogeneous cables, characterizing the onset and development of alternans with standard electric and novel magnetic restitution curves (Crispino et al. 2025; Nicoletti et al. 2025; Tse et al. 2016). Also, we expand our work including the simulation of pseudo-Electrocardiograms (pseudo-ECGs), and the analysis of the corresponding magnetic field, for identifying the typical ST-segment elevation and T-wave inversion characteristic of saddleback and coved type BrS.

## Methods

In this section we provide a description of the mathematical model implemented to simulate the electrical activity of healthy and pathological cells. Next, we describe how the magnetic field was obtained from the simulated electrical activity. Finally, we report technical information regarding the analysis of the simulated data and the numerical aspects of the work.

### Cardiac Electrophysiology Modeling

Cardiac action potentials in healthy and pathological cases were simulated using a phenomenological model of cardiac electrophysiology suitable for reproducing the saddleback and coved type action potentials characteristic of BrS (Bueno-Orovio et al. 2015, 2008; Gizzi et al. 2013), as well as for future extensions of the work including temperature-related effects. The action potential propagation is described through a reaction diffusion partial differential equation (Eq. 1), including the contribution of fast-inward, $J_{fi}$, slow-outward, $J_{so}$, and slow-inward currents, $J_{si}$:

1 $$\begin{aligned} \frac{\partial u}{\partial t}= D\nabla ^2u-(J_{fi}+J_{si}+J_{so}) \,, \end{aligned}$$

where *u* is an variable adimensional representing membrane potential (ranging in 0 - 1.5), and *D* is the diffusion coefficient. $J_{fi}$, $J_{si}$, and $J_{so}$ depend on the membrane potential, *u*, and the gating variables, *v*, *w*, and *s* as stated by the following equations:

2a $$\begin{aligned} J_{fi}&=H(u-\theta _v)(u-\theta _v)(u-u_u)\frac{v}{\tau _{fi}}, \end{aligned}$$

2b $$\begin{aligned} J_{si}&=H(u-\theta _{si})\frac{ws}{\tau _{si}}, \end{aligned}$$

2c $$\begin{aligned} J_{so}&=(1-H(u-\theta _{so}))\frac{(u-u_o)}{\tau _{o}}+\frac{H(u-\theta _{so})}{\tau _{so}}. \end{aligned}$$

where, *H* indicates the Heaviside step function, $\theta _v$, $\theta _{si}$, $\theta _{so}$, $u_u$, $\tau _{fi}$, $\tau _{si}$, $\tau _o$, $\tau _{so}$, and $u_o$ are model parameters listed in the Appendix Table 1. In the following, all the quantities which are not explicitly defined in the text are intended as parameters whose values are listed in the Appendix Table 1.

The gating variables respect the following dynamical system:

3a $$\begin{aligned} \frac{\partial v}{\partial t}&=(1-H(u-\theta _v)) \frac{v_\infty -v}{\tau _v^-}-\frac{H(u-\theta _v)}{\tau _v^+},\end{aligned}$$

3b $$\begin{aligned} \frac{\partial w}{\partial t}&=(1-H(u-\theta _w)) \frac{w_\infty -w}{\tau _w^-}-\frac{H(u-\theta _w)}{\tau _w^+}, \end{aligned}$$

3c $$\begin{aligned} \frac{\partial s}{\partial t}&= \frac{(1+\tanh (k_s(u-u_s)))/2-s}{\tau _s}, \end{aligned}$$

with, $w_\infty$ and $v_\infty$ defined as:

4 $$\begin{aligned} v_\infty= & H(\theta _v^{\infty }-u), \end{aligned}$$

5 $$\begin{aligned} w_\infty= & (1-H(u-\theta _w^{\infty })) \left( 1-\frac{u}{\tau _{w_\infty }}\right) +H(u-\theta _w^{\infty })w_\infty ^\star . \end{aligned}$$

Model equations Eqs. 2a-3c are completed with voltage-dependent time constants:

6a $$\begin{aligned} \tau _v^-&=(1-H(u-\theta _v^-))\tau _{v1}^{-}+H(u-\theta _v^-)\tau _{v2}^{-},\end{aligned}$$

6b $$\begin{aligned} \tau _w^+&= \tau _{w1}^+ +(\tau _{w2}^+ - \tau _{w1}^+) \frac{\tanh (k_w^+(w-w_c^+))+1}{2},\end{aligned}$$

6c $$\begin{aligned} \tau _w^-&= \tau _{w1}^- +(\tau _{w2}^- - \tau _{w1}^-) \frac{\tanh (k_w^-(u-u_w^-))+1}{2},\end{aligned}$$

6d $$\begin{aligned} \tau _{so}&= \tau _{so1} +(\tau _{so2} - \tau _{so1}) \frac{\tanh (k_{so}(u-u_{so}))+1}{2},\end{aligned}$$

6e $$\begin{aligned} \tau _s&=(1-H(u-\theta _s))\tau _{s1}+H(u-\theta _s)\tau _{s2},\end{aligned}$$

6f $$\begin{aligned} \tau _o&=(1-H(u-\theta _o))\tau _{o1}+H(u-\theta _o)\tau _{o2},\end{aligned}$$

6g $$\begin{aligned} \tau _{si}&=\tau _{si1} +(\tau _{si2} - \tau _{si1}) \frac{\tanh (k_{si}(s-s_{c}))+1}{2}. \end{aligned}$$

We study the propagation of electrical signals in a simplified geometry, consisting of a straight 3 cm long 1D cable resembling a cardiac fiber, with a spatial discretization $\Delta z=100~\mu$m. Specifically, we simulate the behavior of healthy myocardial, endocardial and epicardial cells (Bueno-Orovio et al. 2008). Besides, we study pathological epicardial behaviors representative of BrS saddleback and coved patterns (Bueno-Orovio et al. 2015).

In addition, we simulate the electrical activity on a 0.6 cm long cable with a distribution of cells resembling a tract of human right ventricle, thereby containing epicardial, endocardial and myocardial cells distributed according to Bueno-Orovio et al. (2015).

Model parameters are selected according to Bueno-Orovio et al. (2015, 2008) and listed in Table 1 of the Appendix.

### Magnetic field modeling

The propagation of the action potential along the cable drives currents which are the primary source of the magnetic field outside the cell membrane (Roth and Wikswo 1985; Roth et al. 1988).

As established by the classical electromagnetism laws, the current density can be estimated by taking the spatial gradient of the membrane potential, which for a 1D cable reduces to the partial derivative along the $z-$ axis:

7 $$\begin{aligned} J(t,z')=-\sigma \frac{\partial V_m}{\partial z'}. \end{aligned}$$

In Eq. 7, and in the following, primed coordinates are referred to positions within the source (the 1D-cable), while non-primed coordinates will be used for positions in the volume surrounding the cable. The membrane potential $V_m$ is obtained from its dimensionless equivalent, *u*, applying the following transformation: $V_m(t,z')=\left[ 85.7u(t,z')-84\right] ~$mV, where $-84$ mV is the resting potential and 85.7 mV is a scaling factor reflecting the amplitude of the action potential (Bueno-Orovio et al. 2008, 2015). The current density *J* is obtained assuming that the cable is homogeneous and isotropic with constant conductivity estimated as Fenton and Karma (1998) $\sigma =D S_0 C_m$, where,

$D=1.17\cdot 10^{-3}$ cm^2^/ms is the diffusion coefficient, $S_0$ is the surface to volume ratio calculated by modeling cardiac myocytes as cylindrical compartments with $R'=40~\mu$m and $L=100~\mu$m, and $C_m=1~\mu$F/cm^2^ is the membrane capacitance per unit area.

The computed current density is assumed to be uniformly distributed over the section of a cylindrical cable with radius $R'=40~\mu$m, so that the current originating the magnetic field can be written as:

8 $$\begin{aligned} I(t,z')=\int _{S'}\vec {J}(t,\vec {r'})\cdot d\vec {S'}= J_z(t,z')\pi R'^2, \end{aligned}$$

where, $dS'=dx'dy'$ is the infinitesimal element of surface defined on the source. This assumption allows us to compute the magnetic field solving the one dimensional integral of the Biot-Savart’s law (for further details we refer the reader to Crispino et al. (2025)):

9 $$\begin{aligned} \vec {B}=\frac{\mu _0}{4\pi }\int _{z'_{\min }}^{z'_{\max }}\frac{I(t,z')d\vec {z'} \wedge \Delta \vec {r}}{\vert \Delta \vec {r}\vert ^3}. \end{aligned}$$

In Eq. 9, $\Delta \vec {r}= \vec {r}- \vec {r^{~'}}$ with $r=(x,y,z)$ the position in which the magnetic field is evaluated and $\vec {r^{~'}} = (0,0,z')$ the position along the cable. The integral is performed over the entire length of the cable, assumed as a straight cylinder in the *z*-direction with $z'\in [z'_{min},z'_{max}]=[0, 3]$ cm. Taken into account the assumptions explained above, by explicitly solving the cross product in Eq 9 we obtain:

10 $$\begin{aligned} & \vec {B} =- \frac{\mu _0}{4\pi }\int _{z'_{\min }}^{z'_{\max }}\frac{I(t,z')y\,dz'}{[x^2+y^2+(z-z')^2]^{\frac{3}{2}}} \,\hat{i}\nonumber \\ & \quad\quad+\frac{\mu _0}{4\pi }\int _{z'_{\min }}^{z'_{\max }}\frac{I(t,z')x\,dz'}{[x^2+y^2+(z-z')^2]^{\frac{3}{2}}} \,\hat{j}, \end{aligned}$$

where $\hat{i}$ and $\hat{j}$ indicate the *x* and *y* unit vectors, respectively. Therefore, the magnetic field is evaluated solving Eq. 10 in a cylindrical domain ($L=3$ cm, $R=300~\mu$m) surrounding the cable for its entire length.

The surrounding domain is assumed to be a non-conductive medium with magnetic permeability of the vacuum. It is worth noting that, in physiological conditions, the cardiac tissue is immersed in a saline conductive medium rather than in a insulating medium as here assumed. The effect of this conductive environment has to be taken into account while evaluating the magnetic field, as it is the site of secondary ohmic currents driven by the primary hearth activity, which inevitably contribute to the recorded magnetic field (Leifer et al. 1986). However, in our specific case, this assumption do not significantly alters the results. Indeed, the effects of secondary currents in the extracellular environment are negligible at distances from the source smaller than the length of the depolarization wavefront (Wikswo et al. 1979; Tan et al. 1990; Roth and Wikswo 1985; Roth and Woods 1999; Leifer et al. 1986; Tan et al. 1990; Roth et al. 1988). This approximation is verified in our case as we evaluate the magnetic field at $d=50~\mu$m from the source, while the depolarization wavefront extension is around 1 mm.

***Numerical Methods*** Healthy and pathological models of cardiac electrophysiology have been implemented in 1D cables using the finite-difference method for both spatial and temporal derivatives in MATLAB R2022b (MathWorks, Natick, Massachusetts). The electrophysiology is solved implementing the Euler scheme (Teukolsky et al. 1992) and the magnetic field is then computed integrating Eq. 10 using the trapezoidal approximation method. The spatial discretization of the cable and of the surrounding domain, as well as the temporal discretization, have been selected to ensure an accurate computation of the AP and of the corresponding magnetic field. The final geometry consisted of a 3 cm long cable with a spatial grid of $\Delta z=100~\mu$m surrounded by a cylindrical domain with $R=300~\mu$m and $L=3$ cm discretized in the $x-y$ plane with a spatial grid $\Delta x =\Delta y=10~\mu$m and along the *z*-axis with $\Delta z=100~\mu$m. The same discretization scheme for the cable and the surrounding domain was applied in the simulations on the 0.6 cm cable, which was immersed in the cylindrical domain described just above.

### Pacing protocol and electro-magnetic analysis

In this work we analyze the behavior of healthy and pathological cardiac tissue by means of both the standard electrophysiology indicators and a novel indicator: the norm of the magnetic field, which we already established as a valid tool for the detection of cardiac alternans (Crispino et al. 2025; Nicoletti et al. 2025). As the first step, we analyze the electrophysiological properties of healthy and pathological cases characterizing the behavior of the action potential duration (APD) during a pacing down restitution protocol. We deliver a sequence of 15 current pulses with a duration of 10 ms and a fixed time interval between two subsequent stimuli, then the inter-stimulus interval, usually known as pacing cycle length (PCL), is decreased from 1000 ms until the conduction block is reached. For each PCL and for a fixed position along the cable, we selected the last two action potentials of the series and compute the action potential duration thresholding the action potential at 90% of the repolarization. In this way, we obtained the restitution curves characterizing the response of the tissue to stimuli. Beyond the APD_90_, for each PCL we compute the conduction velocity of the signals along the cable, and the maximal value of the time derivative of the AP ($(\partial V/ \partial t)_{max}$). The former is used as a control parameter, while the latter is used as an indicator of the upstroke velocity, which strongly influences the magnetic field (Eqs. 7 and 9). Indeed, for signals traveling at a constant velocity along the cable, the following relation holds:

11 $$\begin{aligned} \frac{\partial V}{\partial t}=-v \frac{\partial V}{\partial z}. \end{aligned}$$

Therefore, the $(\partial V/ \partial t)_{max}$ is directly related to the maximal intensity of the magnetic field originated by the electrical activity.

We obtain the magnetic equivalent of the APD restitution curves reporting the maximal value of the magnetic field norm computed in the same position and for the same two beats considered in the APD analysis at a distance of $50~\mu$m from the cable.

To further expand our analysis, we compute the transmural pseudo-ECG (Bueno-Orovio et al. 2015) for a 0.6 cm long cable resembling the composition of the right human ventricle and compare the results with magnetic field computed for the same configuration.

12 $$\begin{aligned} ECG=\int {\frac{D\vec {{\nabla }} V \cdot \vec {{r}}}{||r||^3} d\vec {{r}} }. \end{aligned}$$

Pseudo-ECG is computed at $50~\mu$m from the cable axis and at 1 mm from the epicardial end of the cable. Accordingly, a pseudo-magnetocardiogram (pseudo-MCG) is obtained by computing the temporal behavior of $B_y$ component in the same point.

## Results

In the following sections we study the case of healthy epicardial, endocardial and myocardial myocytes obtaining the magnetic equivalent of APD restitution curves. Next, we study the electric and magnetic behavior of pathological epicardial cells in the case of two electrical patterns representative of the Brugada syndrome: the coved type and saddleback type. Finally, we combine the models to simulate transmural pseudo-ECGs and pseudo-MCGs in healthy and pathological cases.

### Electro-magnetic behavior of healthy cardiac cells

**Fig. 1:**
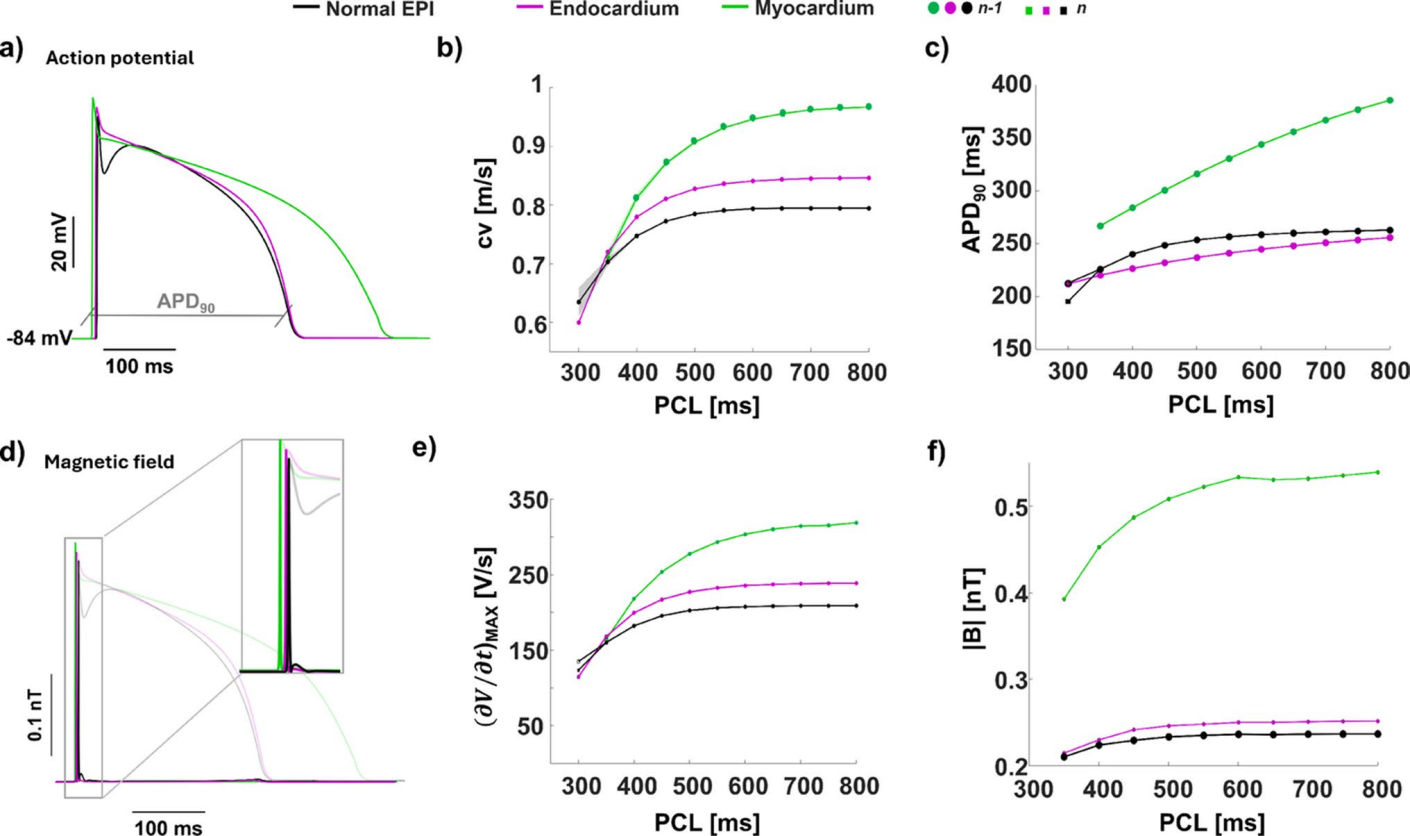
Action potential and magnetic field in healthy epicardium, mid myocardium and endocardium. **a) Epicardial, endocardial and myocardial action potentials**. We show healthy endocardial (in purple), epicardial (in black) and myocardial (in green) action potentials. In gray, a schematic representation of the APD_90_ of endocardial and epicardial action potentials. The three APs are represented for a fixed position along the cable during the stimulation with PCL=1000 ms. **b) Conduction velocity**. In the panel we report the conduction velocity computed for myocardial, epicardial and endocardial action potentials traveling on the cable as a function of the PCL. ${\textbf {c) APD}}_{{\textbf {90}}}$ **restitution curves**. The APD_90_ restitution curve are obtained calculating the APD_90_ for the last two beats of the series in fixed position along the cable and for each PCL. **d) Magnetic field of epicardial myocardial and endocardial cells**. The panel reports the time course of the magnetic field norm generated by epicardial, endocardial and myocardial cells. The representation shows the temporal behavior of the field for a fixed position along the cable during the stimulation with PCL=1000 ms. The shaded curves represent the APs originating the magnetic field. **e)** $(\partial V / \partial t)_{max}$ **restitution curves.** For each PCL of the restitution protocol we report the maximal value of the time derivative of the AP whose duration is reported in panel c), i.e., for each PCL, the last two AP of the series. **f)** $|\textbf{B}|$ **restitution curves.** In the restitution curves we report the maximal value of the magnetic field computed for the beats whose duration and $(\partial V / \partial t)_{max}$ are shown in panels c) and e), respectively. All panels share the same color code: purple for endocardial cells, green for myocardial cells and black for epicardial cells

As the first step, we analyze and compare the electric and magnetic behavior of healthy epicardial, endocardial, and myocardial cells. To this purpose we simulate the electrical and magnetic activity in homogeneous cables composed solely by one type of cells. For each case, we characterize the response of the cells implementing a full pacing-down restitution protocol, reducing the PCL until the conduction block is reached. The simulated epicardial, myocardial, and endocardial action potentials (Fig.1a) are in accordance with previous experimental recordings (Bueno-Orovio et al. 2008; Lukas 1997).

As expected, myocardial action potentials are characterized by longer duration and faster conduction velocity compared to endocardial and epicardial cells (Fig 1a-b, green curves). Also, in agreement with available literature, epicardial action potentials display the classical spike and dome shape and a duration similar to that of endocardial cells (Fig 1a) (Lukas 1997). In epicardial, myocardial and endocardial cables we observe a reduction of the conduction velocity and $(\partial V / \partial t)_{max}$ as the PCL decreases (Fig. 1b,e). APD restitution curves (Fig. 1c) display, at decreasing PCLs, a moderate and slow reduction of the action potential duration in endocardial and epicardial cases (Fig. 1c) (Bueno-Orovio et al. 2008). Myocardial restitution curves are characterized by a steeper decrease compared to the endocardial and epicardial cases (Fig. 1c). Overall, the behavior of the restitution curves for the three cases here studied is in agreement with available experimental data (Bueno-Orovio et al. 2008).

The magnetic field generated by endocardial, myocardial and epicardial cells is linked to the transmembrane potential (Fig. 1d). Although both signals carry equivalent information in a one-dimensional isotropic model, their distinct spatial filtering properties may result in different waveform morphologies and signal-to-noise characteristics, which could be relevant in experimental or clinical settings. The magnetic field produced by endocardial and epicardial cells is characterized by a pronounced peak, rapidly decreasing to zero, in the correspondence of the upstroke and second small peak at the end of the repolarization phase. Epicardial magnetic field is distinguished by an additional peak immediately following the initial peak due to the upstroke. The second peak is peculiarly related to the spike and dome shape of the action potential which affects the temporal behavior of the AP derivative. We quantified the relation between the upstroke velocity and the magnetic field through the corresponding restitution curves (Fig. 1e, f). In particular, the faster is the upstroke velocity, the higher is the intensity of corresponding the magnetic field. In our case, myocardial cells display the fastest upstroke velocity (Fig. 1e), approximately 350 V/s, and therefore they originate the most intense magnetic field (Fig. 1f), around 0.5 nT at 50 $\mu$m from the cable. Epicardial and endocardial cells generate magnetic fields of lower intensities between 0.25 and 0.18 nT. Overall, the magnetic restitution curves reflect the behavior of the $(\partial V / \partial t)_{max}$ curves, decreasing as the PCL is lowered (Fig. 1e).

### Electro-magnetic behavior of epicardial cells for BrS

**Fig. 2:**
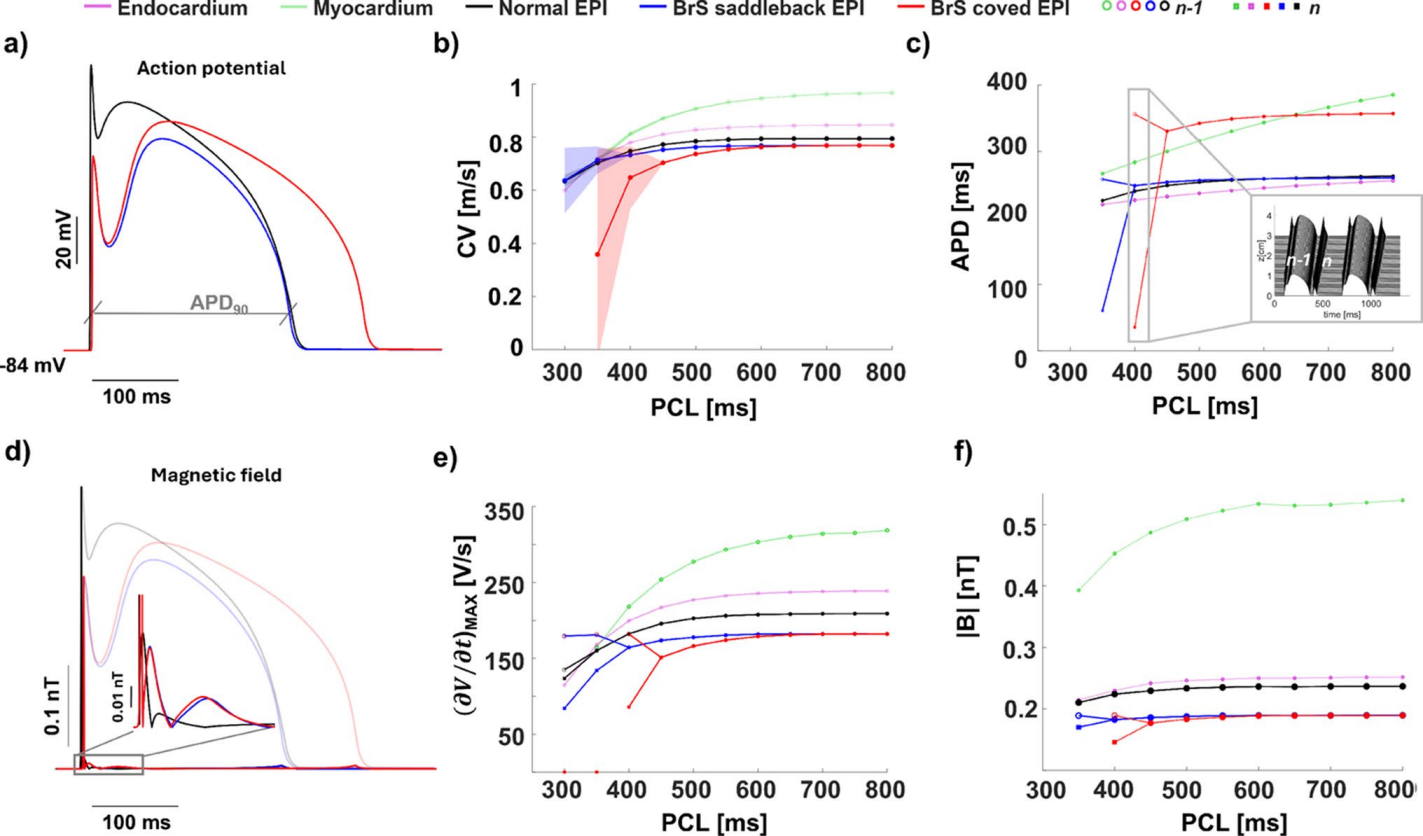
Action potential and magnetic field of Brugada syndrome. **a) Healthy Epicardial, and Brugada syndrome action potentials.** We show healthy epicardial (black), BrS saddleback type (blue) and BrS coved type (red) action potentials. In gray we show a schematic representation of the APD_90_ of normal epicardial action potentials. The three APs are represented for a fixed position along the cable during the stimulation with PCL=1000 ms.** b) Conduction velocity. **In the panel we report the conduction velocity computed for healthy and pathological action potentials traveling on the cable as a function of the PCL.** c) APD**_**60**_** restitution curves.** The APD_90_ restitution curve in healthy and BrS coved and saddleback cases are obtained calculating the APD_90_ for the last two beats of the series in a fixed position along the cable and for each PCL. The insert shows a schematic representation of pathological coved action potentials during alternans regime. **d) Magnetic field of healthy and pathological cases.** The panel reports the time course of the magnetic field generated by normal epicardial, and BrS saddleback and coved action potentials. The representation shows the temporal behavior of the field for a fixed position along the cable during the stimulation with PCL=1000 ms. The shaded curves represent the APs originating the magnetic field. **e) **$\mathbf{(\partial V / \partial t)_{max}}$** restitution curves.** For each PCL of the restitution protocol we report the maximal value of the time derivative of the APs whose duration is reported in panel c), i.e., for each PCL, the last two APs of the series. **f) **$|\textbf{B}|$** restitution curves.** In the restitution curves we report the maximal value of the magnetic field computed for the beats whose duration and $(\partial V / \partial t)_{max}$ is shown in panels c) and e), respectively. All panels share the same color code: black for healthy epicardial cells, blue for BrS saddleback cells and red for BrS coved cells

In this section we describe the electric and magnetic characterization of epicardial cells displaying the characteristic features of the coved and saddleback type BrS. To this purpose, we simulate the magnetic field in homogeneous cables composed of healthy or pathological cells. As usual, we start our analysis from the electrical activity, as it is the source of the magnetic field. Then, we characterize the magnetic signature of the two types of BrS (Fig. 2).

The two characteristic types of APs in BrS have been obtained from the model of normal epicardial cells modifying the parameters affecting the slow inward and fast inward currents, as detailed in Table 1. The saddleback type action potentials are characterized by a reduced upstroke, and, while having an APD similar to normal epicardial cells, they display an enhanced spike and dome feature with a delayed dome development (Fig. 2a). The coved action potentials behave similarly to saddleback ones, exhibiting a short upstroke and a delayed dome. However, in coved APs the dome is significantly prolonged with respect to the normal case, so that the APD is around 350 ms, comparable with that of myocardial cells (Fig. 2a,c and Fig.1a).

As observed previously, in 1D cables composed of normal epicardial cells we do not observe alternans (1c) in APD, CV, or $(\partial V / \partial t)_{MAX}$, and $|\textbf{B}|$. In contrast, alternans appear in both saddleback and coved cases (Fig.2b, c, e, f) and are clearly identified by all the selected indicators. From the electrical point of view, alternans are detectable as beat-to-beat changes in the conduction velocity (Fig. 2b), APD (Fig. 6^2^c), and $(\partial V / \partial t)_{MAX}$ (Fig. 2e). Alternans arise earlier for coved than for saddleback type (PCL=450 ms for coved and PCL=400 ms dor saddleback) and are characterized in both cases by the succession of delayed dome and lost action potentials, as shown in Fig. 2c (insert).

The magnetic field produced by saddleback and coved action potentials is similar to that of normal epicardial cells (Fig. 2d), therefore displaying an initial steep peak due to the upstroke of the AP, a second smaller peak due to the spike-and-dome feature, and a third peak at the end of the repolarization phase (Fig. 2d, insert). Despite the overall similarities, some differences exist between the normal and the two pathological cases. The most remarkable difference is the intensity of the upstroke-related peak, which is significantly reduced in both the BrS types. The second difference is the appearance of an additional small hillock in the $\mathbf {|B|}$ between the second and the third characteristic peaks (Fig. 2d, gray arrow). This hillock might be related to delayed dome features displayed by both the pathological action potentials.

Action potential alternans arising in the pathological cases are, also, clearly reflected in the corresponding $|\textbf{B}|$ restitution curves, which display a bifurcation in correspondence of the alternans onset (Fig. 2f). As expected from the analysis of the $(\partial V / \partial t)_{max}$ restitution curves, at high PCLs saddleback and coved type AP originate the same magnetic field, while, for PCLs below 550 ms, coved action potentials display a faster $|\textbf{B}|$ reduction and start to alternate earlier than saddleback AP, suggesting that in coved type BrS the development of alternans regimes might be facilitated.

### Pseudo ECG and MCG in Brugada syndrome

**Fig. 3:**
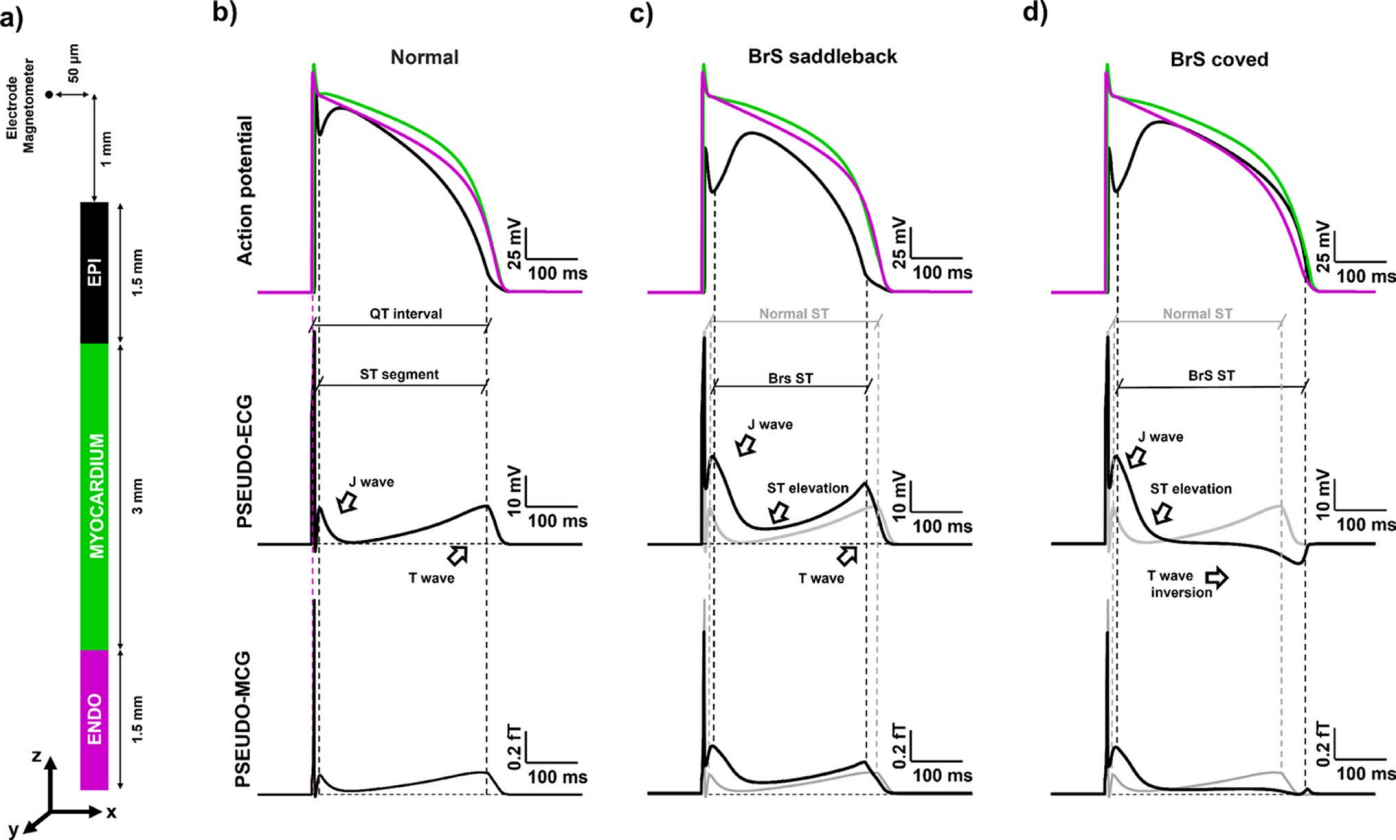
Pseudo-ECG and MCG in health and BrS. **a) Schematic representation of the simulation setup**. The electrical activity is simulated on a 0.6 cm long cable resembling the composition of human right ventricle. **b), c), d) Normal, BrS saddleback and BrS coved action potentials, pseudo-ECGs and pseudo-MCGs** In the top rows we report endocardial, myocardial and epicardial action potentials at three different positions along the cable (top row). In the middle row we report the pseudo-ECG signals generated by the action potentials shown in the top row. In the bottom row we show the $B_y$ component of the magnetic field representing the pseudo-magnetocardiogram. In the middle and bottom row the shaded gray curve represent the normal pseudo-ECG and MCG respectively

As the last step of our study, we expanded our analysis of the magnetic field towards a clinical approach computing pseudo-ECG and pseudo-MCG in 1D cables resembling the human right ventricle structure (Fig. 3).

To this purpose, we distributed along a 0.6 cm long cable endocardial, myocardial, and epicardial cells according to Bueno-Orovio et al. (2015), we stimulate the cable at the endocardial end, and we compute the pseudo-EGC as detailed the Methods 2.3. The pseudo-ECG virtual electrode has been placed 1 mm away from the epicardial end of the cable and at 50 $\mu$m from the cable axis (Fig. 3a). The computed trace is compared with the *y*-component of the magnetic field evaluated in the same position. It is worth noting that, in contrast with the previous analyses, in this case we describe the behavior of a single component of the magnetic field vector, the *y* one, rather than focusing on the norm behavior. Once established that the norm of the magnetic field is a reliable indicator for detecting pathological alterations of the cardiac electrical dynamics (Crispino et al. 2025; Nicoletti et al. 2025), we preferred the description of a single component because of the peculiar need of investigating the signal polarity as a possible indicator of pathological conditions. As usual, we validated our methodology in normal conditions before approaching the disease case. Therefore, we compute the transmural pseudo-ECG for healthy human right ventricle (Fig. 3b), obtaining a waveform in accordance with previous works applying the same methodology (Bueno-Orovio et al. 2015) (Fig. 3b, middle). As expected in our 1D geometry, the magnetic field time course reproduced the characteristics of the pseudo-ECG signal (Fig. 3b, bottom). The only difference regards the QRS complex, whose corresponding peak in the MCG is significantly reshaped. Moving to the case of saddleback BrS (Fig. 3c), the pseudo-ECG shows, in agreement with available literature (Bueno-Orovio et al. 2015; Antzelevitch et al. 2005), that the delayed dome development is mapped in an increased J wave amplitude and in a ST-segment elevation and shortening (Fig. 3c, middle). The J wave enhancement, as well as the the ST segment alterations are clearly reflected by the magnetic field (Fig. 3c, bottom). Finally, pseudo-EGCs of coved-type BrS show a J wave enhancement due to the delayed development of the dome and a the T wave inversion, typical of this form of Brs (Fig. 3c, middle). The T wave inversion, characteristic of coved-type BrS also reflected in the magnetic field, which reflects the features of its source (Fig. 3d, bottom).

## Discussion and conclusion

Developing novel strategies for the non-invasive diagnosis of cardiac diseases is a critical challenge for reducing the mortality due to SCD. In this work we propose an investigation of the electrophysiological alterations due to the Brugada Syndrome through the characterization of the magnetic field originated by the pathological electrical activity.

To this end, we apply our novel methodology for the analysis of the cardiac magnetic activity (Crispino et al. 2025; Nicoletti et al. 2025) to 1D cables composed of healthy human endocardial, myocardial, and epicardial cells, as well as of pathological epicardial cells displaying the features of saddleback and coved type BrS. Our results on normal epicardial, endocardial and myocardial cells show that the magnetic field norm can be used to detect changes in the electrophysiology of the tissue (Fig. 1). The time course of the magnetic field reflects the time course of the time derivative of the action potential, displaying for the epicardial cells an additional peak related to the spike and dome feature characteristic of epicardial cells. Magnetic restitution curves are capable of reproducing $(\partial V / \partial t)_{max}$ restitution curves capturing PCL-dependent changes in the electrical activity of the system.

We demonstrate that the magnetic field analysis might be a valid tool for the investigation of both saddleback and coved type BrS. Magnetic restitution curves of pathological cables show that both types of BrS are prone to develop cardiac alternans which could evolve in to fatal arrhythmias (Fig. 2). In particular, coved type BrS seems to have high propensity to early development of alternans compared to the saddleback type.

In the final part of our study, we simulated transmural pseudo-ECG and pseudo-MCG signals in a 1D cable representing a simplified right ventricular segment. Our results show that magnetic signals are capable of capturing features such as ST-segment elevation, J-wave enhancement, and T-wave inversion associated with Brugada syndrome, in agreement with their electric counterparts.

However, as discussed in literature, in a one-dimensional isotropic model, the electric and magnetic signals are mathematically linked through spatial derivatives, and therefore carry equivalent information. As such, the magnetic field does not provide intrinsically new information with respect to transmembrane or extracellular potentials in this setting. The differences observed in waveform shape or peak timing result from the distinct spatial filtering properties of each signal type.

These differences may still be relevant in practical terms, especially in relation to signal detectability and sensitivity to specific electrophysiological alterations under realistic recording conditions. Nevertheless, a clear informational advantage of the magnetic signal would require conditions such as anisotropy, geometric complexity, or higher-dimensional current patterns, which are not captured by the present 1D model. Future work will therefore aim to extend this framework to two-dimensional domains, where magnetocardiography may reveal features inaccessible to standard electrical recordings (Murdick and Roth 2004; Roth and Woods 1999; Holzer et al. 2004; Roth 1991; Sepulveda and Wikswo 1987; Sepulveda et al. 1989; Roth et al. 1988; Wijesinghe et al. 1991; Wijesinghe and Wikswo 1991; Wijesinghe et al. 1991; Roth and Wikswo 1986; McBride et al. 2010). Similar considerations also hold for the analysis performed in homogeneous endocardial, epicardial and myocardial cables. Summing up, the main limitation of our approach is the simplified geometry and which intrinsically limits the possible scenarios to be investigated. While approaching the discussion of our results it has also to be taken into account that the magnetic signals are intrinsically noisier than the electrical ones, and therefore more difficult to be recorded. The predicted intensities of the pseudo-MCG are in the fT range at 50 $\mu$m from the source, being extremely low to be detected by the state-of-the-art of magnetic field sensors used for biological applications. Nonetheless, it has also to be considered that this work has been performed in an idealized geometry which is not directly reproducible in an experimental context. Future developments of the work will address this limitation by expanding our methodology to more realistic 2D and 3D domains which could be more representative of real experimental samples. Also, we will take into account the role of the extracellular conductive environment and the different degrees of anisotropy in the intracellular and extracellular spaces which both influence the behavior of the magnetic field Murdick and Roth (2004); Roth and Woods (1999); Holzer et al. (2004); Roth (1991); Sepulveda and Wikswo (1987); Sepulveda et al. (1989); Roth et al. (1988); Wijesinghe et al. (1991); Wijesinghe and Wikswo (1991); Wijesinghe et al. (1991)).

In conclusion, in this work we perform an investigation of the magnetic field associated to healthy and pathological cardiac electrical activity in 1D cables. We characterize alternans onset and development with magnetic restitution curves in the case of saddleback and coved type Brugada syndrome. Moreover, we verified, with some limitations, that pseudo-MCG simulations could potentially be applied for detecting pathological alterations due to genetic disorders.

## Appendix

**Table 1:** Model parameters for human epicardial, endocardial and myocardial cells. For epicardial cells we provide the parameters for simulating BrS saddleback and coved types. Model parameters are selected according to Bueno-Orovio et al. (2008, 2015). Units are given in ms, cm, mV, mS, $\mu$F, g. We use the same initial conditions for all simulations: $u=$, $v=1$, $w=1$, $s=0$ (Bueno-Orovio et al. 2008)

Parameter	Normal Epicardial	Brugada saddleback	Brugada coved	Endocardium	Mid Myocardium
$\theta _v$	0.3	0.3	0.3	0.3	0.3
$\tau _{v^-_1}$	60	60	60	75	80
$\tau _{v^-_2}$	1150	100	100	10	1.4506
$\theta _v^-$	0.006	0.006	0.006	0.2	0.1
$\tau _{w^-_1}$	60	60	60	6	70
$\tau _{w_2^-}$	15	15	15	140	8
$k_w^-$	65	65	65	200	200
$u_w^-$	0.03	0.03	0.03	0.016	0.016
$\tau _{w_1^+}$	200	25	25	280	280
$\tau _{w_2^+}$	200	125	180	280	280
$k_w^+$	n/a	5.7	5.7	8	6.5
$w_C^+$	n/a	0.15	0.15	1	0.15
$\tau _{so_1}$	30.0181	30.0181	30.0181	40	91
$\tau _{so_2}$	0.9957	0.9957	0.9957	1.2	0.8
$k_{so}$	22.0458	2.0458	2.0458	2	2.1
$u_{so}$	0.65	0.65	0.65	0.65	0.6
$\theta _{so}$	0.13	0.13	0.013	0.13	0.13
$\tau _{s1}$	2.7342	2.7342	2.7342	2.7342	2.7342
$\tau _{s2}$	16	35	35	2	4
$\theta _s$	0.13	0.13	0.13	0.13	0.13
$u_o$	0	0	0	0	0
$\tau _{o1}$	400	400	400	400	410
$\tau _{o2}$	6	6	6	6	7
$\theta _o$	0.006	0.006	0.006	0.006	0.005
$\tau _{si_1}$	1.8875	7.5476	7.5476	2.9013	3.3849
$\tau _{si_2}$	1.8875	1.8875	1.8875	2.9013	3.3849
$\theta _{si}$	0.13	0.13	0.13	0.13	0.13
$k_{si}$	n/a	6	6	6	6
$s_{c}$	n/a	0.7175	0.7175	1	1
$\tau _{fi}$	0.11	0.04	0.04	0.1	0.078
$\theta _w$	0.13	0.13	0.13	0.13	0.13
$\tau _v^+$	1.4506	1.4506	1.4506	1.4506	1.4506
$k_{s}$	2.0994	5.8	5.8	2.0994	2.0994
$u_{s}$	0.9087	0.35	0.35	0.9087	0.9087
$u_u$	1.55	1.0	1.0	1.56	1.61
$\tau _{w_\infty }$	0.006	0.13	0.13	0.0273	0.01
$w_\infty ^\star$	0.94	0.94	0.94	0.94	0.5
$\theta _v^\infty$	0.006	2	2	0.006	0.1
$\theta _w^\infty$	0.13	0.13	0.13	0.13	0.13
*D* [cm^2^/s]	1.171	1.171	1.171	1.171	1.171
